# Effects of acrylamide on sperm parameters, chromatin quality, and the level of blood testosterone in mice

**Published:** 2014-05

**Authors:** Majid Pourentezari, Alireza Talebi, Abulghasem Abbasi, Mohammad Ali Khalili, Esmat Mangoli, Morteza Anvari

**Affiliations:** 1 *Department of Biology and Anatomy, Shahid Sadoughi University of Medical Sciences, Yazd, Iran.*; 2 *Research and Clinical Center for Infertility, Shahid Sadoughi University of Medical Sciences, Yazd, Iran.*

**Keywords:** *Mice*, *Acrylamide*, *Sperm*, *Chromatin*, *Testosterone*

## Abstract

**Background:** Acrylamide (AA) is an important industrial chemical primarily. AA is also found in carbohydrate-rich foods that are prepared at high temperatures, such as French fries and potato chips. It is demonstrated that AA is a carcinogen and reproductive toxin and has ability to induce sperm damage.

**Objective:** The aim of this study was to observe the effects of AA on sperm parameters and evaluation of sperm chromatin quality and testosterone hormone in mice.

**Materials and Methods: **Totally, 16 adult male mice were divided into two groups. Mice of group A fed on basal diet; group B received basal diet and AA (10 mg/kg, water solution) for 35 days. The right cauda epididymis was incised and then placed in Ham’s F10 culture media at 37^o^C for 15 min. Released spermatozoa were used to analyze count, motility, morphology and viability. To determine the sperm DNA integrity and chromatin condensation, the cytochemical techniques including Aniline blue, Acridine orange and Chromomycin A3 staining were used.

**Results: **AA-treated mice had poor parameters in comparison with control animals. In sperm chromatin assessments, except TB (p=0.16), significant differences were found in all of the tests between two groups. It was also seen a significant decrease in concentration of blood testosterone in AA-treated animals when compared to controls (p<0.001).

**Conclusion:** According to our results, AA can affect sperm parameters as well as sperm chromatin condensation and DNA integrity in mice. These abnormalities may be related to the reduction in blood testosterone.

## Introduction

Acrylamide (CH_2_=CHCONH_2_) (AA) is a widely used industrial chemical which primarily used in the production of copolymers and polymers. Copolymers like Polyacrylamide are used as flocculants in the treatment of sewage and wastewater, as binders in the paper and textile industries, and as soil conditioners (1). AA is found in carbohydrate-rich foods prepared at high temperatures such as potato chips and French fries that are consumed by humans (2). The World Health Organization estimated total daily intake of AA from food in the range of 0.3-0.8 μg/kg of body weight (3). It is shown that AA is a neurotoxic, clastogenic, and mutagenic agent in somatic and germ cells (4-7). AA is considered as a probable human carcinogen (8). 

AA is metabolized by either conjugation with glutathione or oxidation to glycidamide (GA) (9). The formation of GA is mediated primarily by cytochrome P450 2E1 (CYP2E1) (5). Both AA and glycidamide (GA) can bind to hemoglobin and DNA to form adducts (1, 5). Studies confirmed that AA metabolism to glycidamide is a major step in AA-induced mutagen in germ and somatic cell (6, 7). Formation of GA-DNA adducts may be involve in AA-induced toxicity, carcinogenicity, and mutagenicity, (5, 10). AA produces chromosomal aberrations and micronuclei in germ cells as well as sperm head abnormalities in rodents (11, 12). Collins *et al *showed that exposure to AA increases the occurrence of micronuclei in sperm cells of mice and rats (13). There is some evidence indicating that AA is a toxicant for male reproductive system, but very little evidence is available about its toxic effects on the female reproductive system (14). 

The effect of AA on male animals include degeneration of the epithelial cells of the seminiferous tubules in testis, abnormal sperm morphology and reduced sperm count, as a result, decreased fertility rates (15). This toxicity may be attributed to the interfering effect of AA on the kinesin motor proteins that exist in the flagella of sperm resulting in reduced sperm motility (14, 15). Evidence is provided that the clastogenic effects of AA on sperm cells may not be by direct interaction with DNA; instead, these effects may be mediated through interference with the microtubule motor protein kinesin that participates in spindle formation and chromosomal segregation during cell divisions or alkylation of protamines in sperm nucleus (16). 

A new approach to the microscopic assessment of sperm for investigation of male fertility is the evaluation of sperm nuclear chromatin (17). Hence male gamete supplies 50% of the embryonic genome, any anomalies in sperm chromatin can affect embryonic development. It is generally accepted that there is a clear relationship between sperm chromatin/DNA damage and reproductive outcomes (18). Furthermore, sperm chromatin condensation has a key role in male fertility, early embryonic growth and pregnancy results (19). In the process of spermatogenesis, the extent of sperm chromatin compaction changes deeply when histones are replaced in a stride mode by testis-specific nuclear proteins, transitional proteins and finally by protamines. Each anomalies during expression of sperm-specific nucleoproteins change sperm chromatin structure and may cause male infertility (18). 

Inter and intra-molecular disulphide bonds of protamine molecules are crucial for sperm nuclear compaction and stabilization. It is believed that this kind of nuclear compaction protects sperm genome from external damages include oxidative stress, temperature height and acid-induced DNA denaturation (20). Despite the fact that AA by virtue of its metabolism to GA can induce base mutations in somatic cells and cause genetic damage to sperm cells through clastogenic effects, it is still not clear whether AA has direct effects on sperm DNA and chromatin condensation. The present study aims to investigate the effects of AA on sperm parameters, DNA and chromatin remodeling and testosterone level in mice.

## Materials and methods

**Animals and treatments**

In this experimental study, 16 adult male Syrian mice with 50 gr weight and 10 weeks old were divided into two groups. Group A served as controls and fed on basal diet, group B received basal diet and AA (10 mg/kg), dissolved in the drinking water and they were held in cages in a controlled environment (21). Chemical analysis has shown that AA remains stable in water for 1 week. Water consumption per cage was measured during the test to estimate the amount of AA per kilogram of body weight in each mouse. 

The experimental project in was approved by ethics committee of Shahid Sadoughi University of Medical Sciences, Yazd, Iran. During the course of this experiment, we followed the recommendations by our Institutional Animal Care and Use Committee for the handling, maintenance, treatment, and killing of the animals.

**Blood sampling**

After 35 days (one duration of spermatogenesis in mice is about 32 days), heart blood samples were taken and analyzed by mouse testosterone ELISA kit for the quantitative determination of testosterone in mouse serum for each animal. 

**Epididymal sperm aspiration and sperm analysis**

After 32 days, the mice were killed by cervical dislocation. A small part of the caudal epididymis of each mouse was dissected and located in 1 mL of pre-warmed Hams F10 medium. Gentle tearing of the tissue was done to make spermatozoa swim out into the culture medium. The dishes were placed in the incubator for 15 minutes at 37^o^C, 5% CO_2_ for further analysis. Sperm motility, normal morphology, viability and count were evaluated for 200 sperm from each animal. Sperm motion analysis was performed via Makler chamber and light microscopy (Olympus Co., Tokyo, Japan). 

Motility was categorized as proportion of progressive (fast and slow) and non-progressive spermatozoa. In addition, the percentage of sperm cells with normal morphology was obtained by Papanicolaou staining and light microscopy at ×1000 magnifications. Viability was measured by Eosin test for 200 spermatozoa per mice (19, 22).

**Sperm chromatin/DNA evaluation**

DNA integrity and chromatin condensation assessments were done by standard cytochemical techniques including AOT, AB, TB and CMA3 (23). All dyes and chemicals were purchased from Sigma Aldrich Company (St Louis, MO, USA).

**Aniline blue (AB) staining**

Aniline blue selectively stains lysine-rich histones and has been used for the purpose of those sperm chromatin condensation anomalies that are related to residual histones. To do this staining, air-dried smears were prepared from washed semen samples and then fixed in 3% buffered glutaraldehyde in 0.2 M phosphate buffer (pH=7.2) for 30 min at room temperature. Each smear was stained with 5% aqueous AB stain in 4% acetic acid (pH=3.5) for 7 min. In light microscopic evaluation, 200 spermatozoa were counted in different areas of each slide using ×100 eyepiece magnification (18). In this staining, the percentages of unstained or pale blue stained (normal spermatozoa) and dark blue stained (abnormal spermatozoa) will be reported.

**Toluidine blue (TB) staining**

Toluidine blue is a metachromatic dye which determines both the quality and the quantity of sperm nuclear chromatin condensation/DNA fragmentation via binding to phosphate groups of DNA strands (22). Briefly, air-dried sperm smears were fixed in fresh 96% ethanol-acetone (1:1) at 4^o^C for 30 min and then hydrolyzed in 0.1 NHCl at 4^o^C for 5 min. after that, the slides were rinsed 3 times in distilled water for 2 min and in the end stained with 0.05% TB in 50% citrate phosphate for 10 min at room temperature. In each sample, at least 200 spermatozoa were counted under light microscopy using ×100 eyepiece magnifications (18, 23). In TB staining, the chromatin quality of sperm will be assessed according to metachromatic staining of sperm heads in following scores: 0, light blue (good chromatin); 1, dark blue (mild abnormal chromatin); 2, violet; and 3, purple (severe chromatin abnormality). So, the sum of spermatozoa with score 1, score 2 and score 3 is considered as TB^+^ or sperm cells with abnormal chromatin.

**Acridine orange test (AOT)**

Acridine orange is a metachromatic fluorescence probe for demonstration of degree of sperm nuclear DNA susceptibility to in-situ acid-induced denaturation by distinction between native double-stranded DNA (green fluorescent) and denatured single-stranded DNA (red fluorescent). Briefly, the air-dried smears were fixed in Carnoy’s solution (methanol/ glacial acetic acid, 3:1) at 4^o^C for at least 2 hrs. 

Each sample was stained by freshly prepared AO (0.19 mg/ml in McIlvain phosphate-citrate buffer (pH= 4) for 10 min. Smears were assessed on the same day using fluorescent microscope (Zeiss Co., Jena, Germany) with a 460-nm filter (18). For AOT, the percentages of green (normal double-stranded DNA) and orange/red (abnormally denatured DNA) fluorescence spermatozoa per sample will be calculated. 

**Chromomycin A3 (CMA3) staining**

Chromomycin A3 is fluorochrome specific for guanosine cytosine-rich sequences and is used for estimation of the degree of protamination of sperm chromatin (20). For this purpose, the smears were dried first and then fixed in Carnoy’s solution at 4^o^C for 10 min. The slide was treated with 150 µl of CMA3 (0.25 mg/ml) in McIlvain buffer for 20 min. After staining in darkroom, the slides were washed in buffer and mounted with buffered glycerol. In each sample, at least 200 spermatozoa were counted under fluorescent microscope with a 460-nm filter and ×100 eyepiece magnifications. In CMA3 staining bright yellow-stained chromomycin-reacted spermatozoa (CMA3+) are considered as abnormal form and yellowish green-stained non-reacted spermatozoa (CMA3-) are considered as normal form.

**Statistical analysis**

Statistical analysis was performed by SPSS software (Statistical Package for the Social Sciences, version 18.0, SPSS Inc, Chicago, Illinois, USA). Student’s *t*-test was applied to evaluate the data and the term ‘statistically significant’ was used to signify a two-sided p<0.05 for sperm parameters and cytochemical tests.

## Results

**Figure 1:**
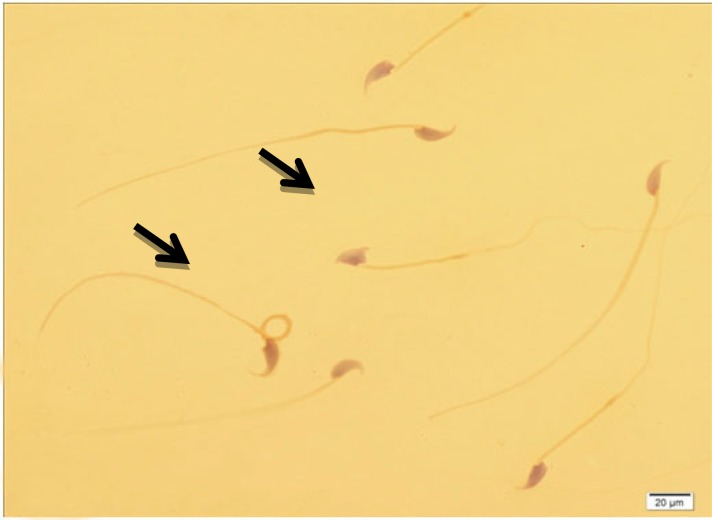
Different forms of sperm morphological abnormalities. The arrow indicates abnormal spermatozoon. Papanicula staining (×100 eyepiece magnification).

**Figure 2:**
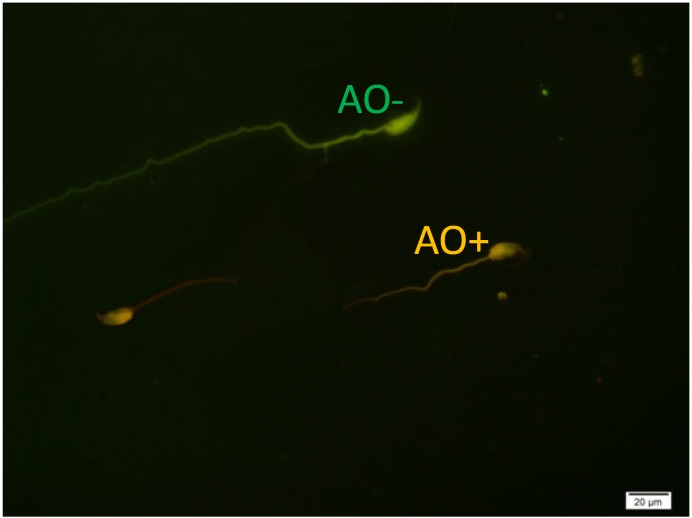
Spermatozoa with native double-stranded DNA (AO-) and denatured DNA (AO+). Acridine Orange staining (×100 eyepiece magnification).

**Figure 3:**
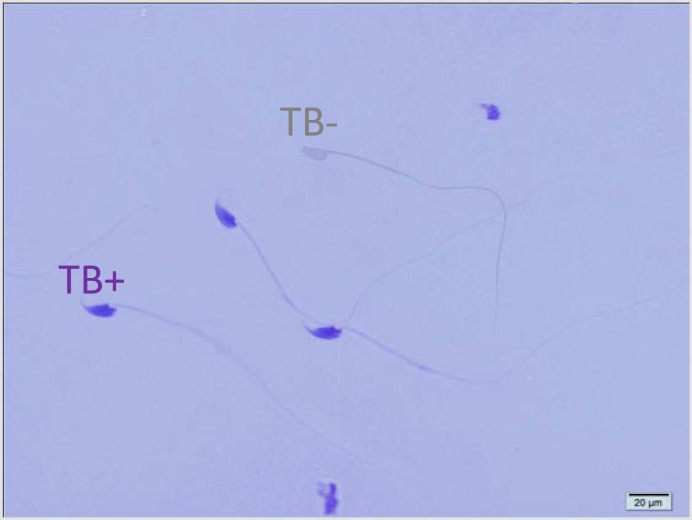
Toluidine Blue staining of spermatozoa. TB+ indicates sperm cells with abnormal chromatin and TB- indicates sperm cells with normal chromatin (×100 eyepiece magnification).

**Figure 4:**
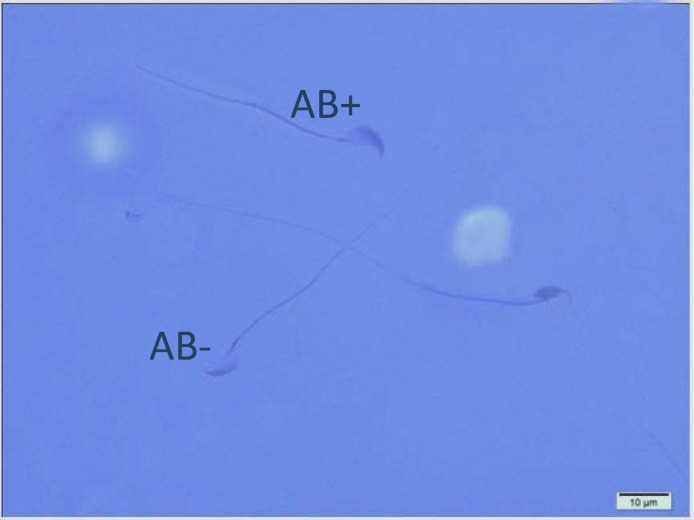
Two Aniline Blue-reacted spermatozoa (AB+) and one normal sperm cell (AB-).Aniline Blue staining (×100 eyepiece magnification).

Table I shows the means and statistical analysis of the various sperm parameters in two groups. It reveals that sperm count, rapid and total motility, morphology and viability were significantly different between groups A and B (see also figure 1). Table II shows the results of analysis of sperm chromatin and DNA integrity (see also figures 2-4). Regarding to AOT, AB and CMA3 staining, we saw statistically significant differences between two groups, but in TB staining, the results didn’t show any significant differences (p=0.161). 

The concentration of testosterone in group B (1.13±1.04) had a significant reduction (p=0.015) when compared with group A (3.25±1.582).

**Table I:** The results of sperm analysis in control (group A) and acrylamide-treated mice (group B)

**Variables**	**Group A**	**Group B**	**p-value**
Count (×106)	110 ± 17.49	90.12 ± 10	0.015*
Rapid motility (%) (Grade a)	20.75 ± 3.01	16.25 ± 3.49	0.038*
Slow motility (%) (Grade b)	23.25 ± 4.86	14.37 ± 3.66	0.003*
Non progressive motility (%) (Grade c)	32 ± 4.37	18.12 ± 5.54	0.001*
Immotile sperm (%) (Grade d)	24 ± 4.27	50 ± 7.69	<0.001*
Total motility (%) (Grade a, b, c)	76 ± 4.27	50 ± 7.69	<0.001*
Normal morphology (%)	75.87 ± 6.72	67 ± 8.01	0.038*
Viability (%)	78.12 ± 5.08	66.5 ± 5.55	0.001*

Data are presented as mean±SD.

* Statistically significant (Student’s *t* test), p<0.05.

**Table II:** The results of sperm chromatin/ DNA evaluation in control (group A) and acrylamide-treated mice (group B)

**Variables**	**Group A**	**Group B**	**p-value**
AO	8.375 ± 3.02	12.625 ± 3.02	0.021*
TB	20.5 ± 2.39	23.75 ± 6.181	0.161
AB	22.5 ± 4.72	28.75 ± 4.267	0.021*
CMA3	3.25 ± 1.669	22.125 ± 4.086	<0.001*

Data are presented as mean±SD.

AO: Acridine orange, TB: Toluidine blue, AB: Aniline blue, CMA3: Chromomycin A3.

* Statistically significant (Student’s *t* test), p<0.05.

**Figure 5:**
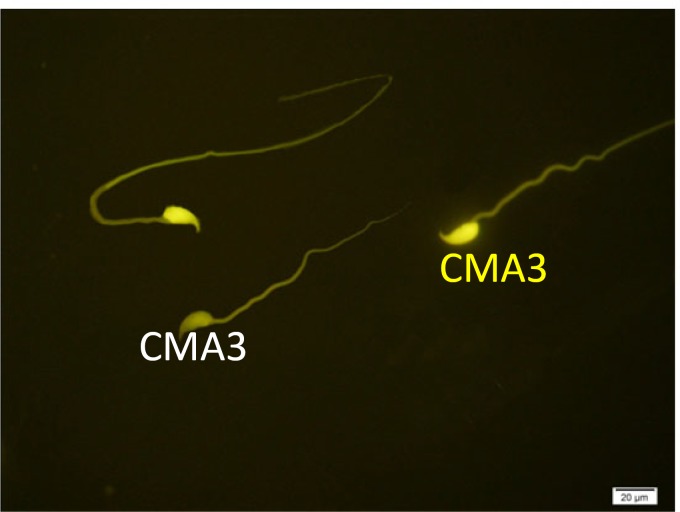
Two spermatozoa with protamine deficiency (CMA3+) and one sperm with normal protamine content (CMA3-). Chromomycin A3 staining (×100 eyepiece magnification).

## Discussion

There are several experimental studies indicating the detrimental effects of AA on male fertility indices including sperm parameters. However, to our knowledge there are few surveys on the impacts of AA on sperm nuclear maturation. In fact, our results have novel information regarding the impacts of AA on sperm chromatin condensation and DNA integrity in mice. Our results indicated that the sperm parameters had statistically significant reduction in AA-treated mice in comparison with controls. Although, Tyl *et al* did not see any significant reduction of sperm motility and concentration in AA-treated rats, more recently, under the same experimental design, Yang *et al* showed a reduction in concentration of epididymal spermatozoa, as well as an increase in morphological abnormalities of spermatozoa resulted by AA in rats (15, 24). 

Regard to sperm count, our result is also in agreement with the study of Wang *et al*. They showed that AA can decrease the concentration of epididymal spermatozoa and may increase histopathological lesions in rat testes (25). Song *et al* observed that chronic exposure to AA can affect sperm development, in particular, an increase in abnormal morphology and decrease in sperm vitality. On the other hand, they showed that AA directly damages Leydig cells and affects the endocrine function of the testis and the process of spermatogenesis (26). Histopathological injuries in the testes such as swelling, vacuolation, necrosis of the spermatids, increase apoptotic cells, and formation of multinucleated giant cells in the seminiferous tubules are considered as the other changes in the cases of AA consumption (24). 

Our previous study in experimental model also showed that following administration of different doses of AA, the viability of sperm cells well decrease. In fact, the functional intact membrane of the sperm tail in both low and high-dose groups had a significant reduction, but membrane integrity of the sperm heads decreased significantly only in the high-dose group (21). It should be noted that high doses of AA cause endothelial thickening of seminiferous tubes, which consequently reduce sperm production (24). In present study, the sperm chromatin quality was another parameter which was compared between two groups. We showed that during the process of spermatogenesis, AA can affect sperm chromatin remodeling and production of sperm cells with less condensed nuclei. It is generally accepted that the occurrence of any anomaly in testicular expression and/or incorporation of every category of sperm-specific nucleoproteins may change sperm chromatin structure and will cause male infertility (18). 

In AB staining which is specific for sperm excessive histones, we found a significant difference between groups. So, it can be say that the AA had detrimental effects on histone-protamine’s replacement during the testicular phase of sperm chromatin packaging. To our knowledge, this is the first report on using of AB staining in assessment of sperm chromatin following AA treatment. In TB staining, although we saw a difference in percentage of TB reacted spermatozoa between two groups, but it was not statistically significant. As it was mentioned before, TB is a metachromatic bye which can bind to phosphate groups of DNA backbone and demonstrates DNA fragmentation. So, according to our results, AA doesn’t have any sever effects on sperm DNA fragmentation in mice. To compare our data to other findings, we didn’t see any similar study in literature. On the other hand, in AO test, we saw a prominent difference between two groups. In CMA3 staining which is specific for sperm nuclear protamine deficiency, we saw a significant difference in percentage of CMA3-reacted spermatozoa between AA and control groups (18). 

The reduction of serum testosterone in AA-treated mice was another finding of our study. It was found by Yang *et al* that testosterone concentration decreases in the serum of AA-treated rats. They also concluded that AA can reduce the viability of Leydig cells, which in turn, diminishes spermatogenesis in the rat testis (24). Conversely, hyperplasia of Leydig cells associated with germ cell loss was reported in mice exposed to AA (27). In fact, the Leydig cells regulate tubular function and produce the high local concentrations of testosterone and control spermatogenesis (28). Therefore, the hyperplasia of Leydig cells may affect male reproductive function.

There are several mechanisms to explain the etiology of reproductive toxicity of AA. At first, it should be noticed that AA, by its effects on Leydig cells and seminiferous tubules and by increasing perturbed gene expression level, causes histopathological changes in the testis and decreases serum testosterone and spermatogenesis which affects sperm parameters including sperm count, motility, normal morphology and viability. Mutagenicity is considered as the second possible mechanism of AA. 

There are several studies on the induction of dominant lethal mutations by this chemical compound. In fact, it is known as a germ cell mutagen which induces clastogenic effects mainly in spermatids of mice and rats (11, 16). AA is metabolized by hepatic P450 CYP2E1 into GA (9). It is shown that approximately 50% of orally administered AA is metabolized via this pathway and produced GA binds to DNA and induces DNA adducts and mutations (29). Indeed, GA is known to be clastogenic and mutagenic both in vitro and in vivo (30). Sega and Generoso observed that exposure to AA can cause DNA breakage during specific stages of germ cell development in male mice (31). In another study, AA has been reported to possess clastogenic and mutagenic properties in vivo (32). 

Furthermore, it had been shown that AA can induce a significant increase in chromatin damages in liver, spleen, and testes diploid cells after treatment (33). Another explanation for the effects of AA has been revealed by Tyl and Friedman (14). They showed that AA and/or GA bind to spermatid protamines and cause dominant lethality and has detrimental effects on sperm morphology. This chemical compound also binds to the motor proteins including kinesin and dynein, resulting in reduction of sperm motility by interference with flagellar function. Of course, the attachment of AA to nuclear protamines may be a good explanation for reducing sperm chromatin condensation in our experimental group. 

Finally, the increase in reactive oxygen species production and decrease in antioxidant capacity of germ cells is considered as another mechanism of AA actions. It is shown that AA through induction of oxidative stress decreases the quality of spermatocyte spermatogonia in mouse testis. In addition, this chemical also reduces peroxide dismutase and glutathione peroxidase enzymes which are two main antioxidants of semen (34). 

## Conclusion

In conclusion, our study showed that AA can affect sperm parameters as well as sperm chromatin condensation and DNA integrity in mice. However, these abnormalities may be related to the reduction of blood testosterone in AA-treated mice.

## Conflict of interest

The authors declare that there is no conflict of interest.

## Footnote

***This article extracted from M.Sc. thesis. (Majid Pourentezari)***
